# Finite Element Analysis on the Effect of Activation Time on Cervical Spine Biomechanical Response During Pilot Ejection

**DOI:** 10.1155/abb/7484728

**Published:** 2026-04-30

**Authors:** Jiongxiang Zhao, Zanni Zhang, Zsolt Radak, Xuanzhen Cen, Ee-Chon Teo, Minjun Liang, Yaodong Gu

**Affiliations:** ^1^ Department of Radiology, Ningbo No. 2 Hospital, Ningbo, China, nbws.gov.cn; ^2^ Faculty of Sports Science, Ningbo University, Ningbo, China, nbu.edu.cn; ^3^ Research Center for Molecular Exercise Science, Hungarian University of Sports Science, Budapest, Hungary, uni-mate.hu; ^4^ Faculty of Engineering, University of Szeged, Szeged, Hungary, u-szeged.hu

**Keywords:** cervical spine biomechanics, finite element analysis, high-g acceleration, injury prevention, muscle activation time, pilot ejection, spinal stability

## Abstract

This study uses finite element analysis (FEA) to investigate different muscle activation times on the cervical spine biomechanical responses in pilot ejection. A validated C0–T1 cervical spine model, incorporating vertebrae, intervertebral discs, ligaments, and 13 major active muscles, was subjected to simulated ejection conditions of 10G vertical acceleration over 150 ms. Activation times ranging from 26 to 92 ms were analyzed to evaluate their impact on vertebral rotation, disc stress, and injury risk. Results demonstrated that shorter activation times (26–46 ms) reduced excessive flexion at C3–C4 and C4–C5; these earlier muscle engagements enhance spinal stability. Conversely, longer delays (76–92 ms) increased rotational angles at C5–C7, exacerbating hyperflexion‐related injury risks. An optimal activation time of approximately 46 ms minimized flexion without inducing compensatory hyperextension, balancing load distribution across cervical segments. These findings emphasize the critical role of neuromuscular response timing in mitigating cervical spine injuries during high‐G ejection. The study provides insights for optimizing pilot safety through tailored muscle activation strategies, sophisticated artificial intelligence (AI) protective equipment design, and training protocols.

## 1. Introduction

In humans, the cervical spine is a highly exposed, unprotected, flexible, and complex biomechanical structure composed of seven vertebrae (C1–C7), intervertebral discs, ligaments, and muscles, which together provide both mobility and structural stability to the head–neck system. Under coordinated neuromuscular actions, it plays a crucial role in maintaining head orientation, supporting vision, and absorbing external mechanical forces [1]. However, due to its anatomical configuration and biomechanical properties, the cervical spine is particularly susceptible to injuries, especially in high‐acceleration environments such as vehicle collisions, sports impacts, and emergency ejection scenarios [2, 3]. Among these, pilot ejection from high‐speed aircraft presents one of the most extreme loading conditions for the cervical spine, often resulting in excessive rotational motion and high mechanical stress, which can lead to severe injuries [4, 5].

During an aircraft emergency ejection, the pilot experiences extreme acceleration forces as the ejection seat propels them away from the aircraft in a fraction of a second [6]. The pilot can experience a vertical acceleration of 10–15G with an acceleration rate of up to 125G/s in a short duration of ~150 ms. In such extreme conditions, the cervical spine undergoes short impulsive flexion–extension motions, leading to large intersegmental rotations between adjacent vertebrae. This excessive motion can result in a range of injuries, including vertebral fractures, ligamentous tears, intervertebral disc herniation, and even spinal cord damage [7–9]. Several studies have highlighted that high‐G acceleration events during ejection induce substantial mechanical loads on the cervical spine, often exceeding physiological tolerance levels, thereby increasing the likelihood of acute injury and long‐term musculoskeletal disorders [10–13].

The cervical spine’s response to external forces is influenced by passive and active biomechanical components [14, 15]. Passive components include intervertebral discs, ligaments, and facet joints, which provide structural integrity and limit excessive motion. These structures play a vital role in stabilizing the cervical spine under normal physiological conditions, but they may not be sufficient to withstand the extreme forces encountered during pilot ejection [16]. In contrast, active components, such as neck muscles, contribute significantly to dynamic stability by actively controlling vertebral motion and absorbing impact forces [17, 18]. The sternocleidomastoid, trapezius, semispinalis capitis, splenius capitis, and scalene muscles are the main cervical muscle groups that work to prevent the head and neck from excessive flexion/extension motion. Research suggests that coordinated activation, either bilaterally or unilaterally, of these muscles helps to mitigate the risk of cervical spine injuries by reducing vertebral rotations and lowering mechanical stress on intervertebral discs [19, 20]. However, the effectiveness of these muscles in preventing injuries depends largely on the forces/turning moment generated at each vertebral level, which are closely related to muscle activation time and muscle structural/mechanical characteristics.

One of the most critical factors affecting cervical spine biomechanics during ejection is muscle activation time (also referred to as reflex delay time), which represents the time required for neck muscles to contract in response to an external stimulus. This activation time is influenced by neural conduction velocity, muscle fiber composition, and motor control mechanisms [21]. In general, human neck muscles exhibit reflex delays ranging from 26 to 92 ms, depending on the type of muscle group and the intensity of the external perturbation [22]. During pilot ejection, the delay in muscle activation can significantly affect the cervical spine’s ability to resist excessive motion [10]. In a delayed activation time (e.g., >76 ms), the cervical spine may undergo excessive flexion or extension motions before the muscles generate sufficient force/moment to counteract these motions. This delayed response could lead to higher intervertebral stress, increased ligament strain, and lead to likelihood of cervical spine injuries [19, 23]. Conversely, in shorter activation times (e.g., 26–46 ms), early muscle actions stabilize the cervical spine, reduce peak rotation angles, and prevent excessive joint loading. However, if the activation is too rapid or too strong, it may induce unintended joint loading effects, potentially causing excessive compressive or shear forces on intervertebral structures [24].

Despite earlier research on the cervical spine’s biomechanics under varied loading scenarios, there are still a number of important unanswered questions about the function of muscle activation time in pilot ejection scenarios [25, 26]. Previous studies mainly compared the presence or absence of muscle activation or simplified reflex conditions. However, the influence of a wide range of physiologically plausible activation delays on cervical kinematics during ejection has not been systematically investigated. Traditional experimental methods, such as cadaveric studies and in vivo measurements, provide valuable insights but with inherent limitations, including ethical concerns, limited sample sizes, and difficulties in replicating extreme ejection conditions [27]. Computational methods, particularly finite element analysis (FEA), have emerged as powerful tools for simulating complex biomechanical interactions in the cervical spine [28]. FEA enables researchers to simulate different ejection conditions, test various muscle activation strategies, and analyze the corresponding kinematic and kinetic responses of the cervical spine [29]. By incorporating muscle activation timing as a variable, finite element (FE) models can provide quantitative insights into how different activation times influence vertebral motion, disc stress, and ligament loading during high‐G events. These findings can contribute to the development of injury prevention strategies, pilot training programs, and enhanced protective equipment designs.

Using a FE model, this study attempts to examine various muscle activation durations on the cervical spine’s biomechanical responses in simulated ejection. Accordingly, in this study: (1) different activation times of 26, 36, 46, 56, 66, 76, and 92 ms on the cervical vertebrae rotation, intervertebral disc stress, and ligament strain during ejection are computed; (2) determination of optimal activation time that minimizes excessive cervical spine motion and reduces the risk of neck injuries; and (3) evaluation of individual contribution of key cervical muscle groups, in stabilizing the cervical spine under ejection conditions. By tackling these goals, the study hopes to advance our understanding of cervical spine biomechanics and aid in the creation of better neck protection techniques for pilots in high‐G emergency ejection.

## 2. Materials and Methods

From an engineering perspective, muscles can be thought of as nervous system‐controlled actuators. All muscle tissues exhibit distinct behavioral characteristics of extensibility (capacity to be stretched or to grow longer), elasticity (capacity to return to its original length after being stretched), irritability (capacity to react to a stimulus), and tension‐development capacity. There are two main parts to muscle’s elastic activity. First, when a muscle is passively stretched, its parallel elastic component (PEC), the muscle membranes, acts and resists. Second, for a tight stretched muscle, its series elastic component (SEC) consists mainly of the tendons, acts like a spring, and stores elastic energy. Muscles stretch and recoil in a time‐dependent manner due to the viscous nature of its components. Muscles also generate tension in reaction to a stimulus as a result of its irritability capacity. The contractile component (CE) of muscle has historically been used to characterize the active tension/force magnitude in terms of muscle length, force‐deformation and force–time functions. This contractile feature makes it possible for muscles to generate active tension to carry out the crucial tasks of moving the body’s limbs, absorbing trauma, and maintaining an upright position [30].

### 2.1. FE Model

A C0–T1 FE model, previously developed and validated by Zhang et al. [31], was used in this study to simulate the biomechanical response of the cervical spine during pilot ejection. The model was constructed based on anatomical information obtained from cadaver specimens and CT scans, providing a geometrically accurate representation of the cervical spine. The validation of the model included comparisons of predicted moment‐rotation relationships of cervical motion segments under physiological static loading conditions, as well as dynamic loading scenarios, such as near‐vertex drop impact and rear‐end impact (whiplash). The simulated responses showed good agreement with previously published experimental data, demonstrating the model’s capability to reproduce the basic biomechanical behavior of the human cervical spine under both static and dynamic loading conditions.

The accurate anatomical model included all critical vertebrae (C0–T1), intervertebral discs, associated ligaments, articulated facet joints, and neck major muscles. The bony cortical and cancellous bones and intervertebral discs are modeled as solid elements with appropriate elastic properties. Main ligaments, such as anterior longitudinal ligament (ALL), and posterior longitudinal ligament (PLL) modeled as link elements were included to increase the model’s mechanical response’s accuracy under dynamic loading situations. Using ANSYS R16 and LS‐DYNA solvers, parameters defining the active characteristics of the major neck muscles were incorporated in the ejection simulation analysis [32–38].

**Figure 1:**
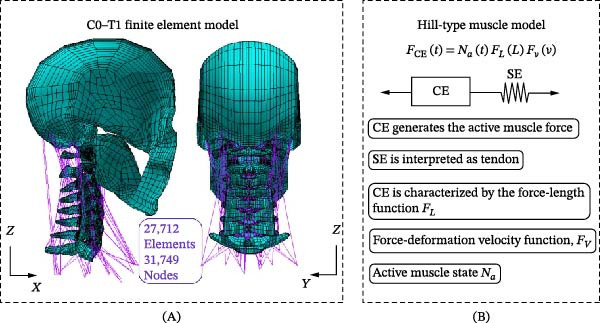
(A) Lateral and posterior views of the C0–T1 FE model and (B) the Hill‐type muscle model.

The model consists of 31,749 nodes and 27,712 elements (Figure 1A). Based on published literature, various 2D and 3D element types with distinct mechanical and structural properties were assigned to characterize the bony vertebrae, ligaments, intervertebral discs, and muscles [34, 39–42] as listed in Table 1. The previous validated FE C0–C7 cervical spine model a physiological lordotic curvature of ~37°, consistent with the neck posture of a seated 50th percentile male [31, 43]. Additionally, the head center of gravity (CG) was defined based on published biomechanical data. Mass elements were assigned to nodes near the CG of the skull in the midsagittal plane to represent a head mass of 5.5 kg and a corresponding moment of inertia of 0.035 kg·m^2^ [30, 31]. The relative positions of the head, occipital condyles (OC), and T1 vertebra follow the neutral anatomical alignment commonly used in validated cervical spine models. The initial orientations of the vertebrae were defined to reproduce the physiological cervical lordosis in the sagittal plane. As this study emphasized on time activation of muscles, Section 2.2 provides brief description on modeling of muscles.

**Table 1:** Material properties for various components in the FE model.

Component	Young’s modulus (MPa)	Poisson’s ratio	Density (kg/mm^3^)
Cortical bone	12000.0	0.29	1.83E‐06
Cancellous bone	450.0	0.29	1.00E‐06
Endplates	500.0	0.40	1.83E‐06
Posterior element	3500.0	0.29	1.83E‐06
Annulus	3.4	0.40	1.20E‐06
Nucleus	1.0	0.49	1.36E‐06
Ligaments			
ALL	30.0	0.30	—
PLL	20.0	0.30	—
ISL, LF	10.0	0.30	—
SSL	1.5	0.30	—
CL(C0–C1)	1.0	—	—
CL(C1–C3)	10.0	0.30	—
CL(C3–C7)	20.0	—	—
AlL	5.0	0.30	—
TL	20.0	0.30	—
ApL	20.0	0.30	—
AM	20.0	0.30	—
PM	20.0	0.30	—
NL	20.0	0.30	—

Abbreviations: ALL, anterior longitudinal ligament; AlL, alar ligament; AM, anterior membrane; ApL, apical ligament; CL, capsular ligament; ISL, inter spinous ligament; LF, ligamentum flavum; NL, nuchal ligament; PLL, posterior longitudinal ligament; PM, posterior membrane; SSL, supra spinous ligament; TL, transverse ligament.

### 2.2. Active Muscle Modeling

To model muscle, its’ contractile characteristics of the fibers are modeled either in parallel or in series with muscle elastic component. In 1938, Hill [44] created the first comprehensive model to define muscle force. A simplified Hill‐type muscle model, which consists of the contractile element (CE) in series with an elastic element (SE), is shown in Figure 1B. In clinical, athletic, and occupational biomechanics, numerous researchers have created their own numerical models based on Hill’s model to forecast muscle forces while simulating human body movements [14, 45–50]. However, there are limited studies on the utilization of these muscle models in FEA of head–neck complex under simulated impact. Currently, the commonly used FEM software (ANSYS, ABACUS, etc.) does not have standard element type and material properties to describe the complex tension generated in activated muscle. The muscles around the cervical spine column were typically simplified as spring or spring‐damper elements in the numerical studies that were available and focused on the dynamic response of the head–neck complex [27].

The C0–T1 FE model incorporated 12 key cervical muscle groups essential for the head/neck mechanics. All these muscles either shorten or elongate bilaterally/unilaterally in isometric or isotonic manners, resulting in flexion/extension with different articulation angulation motions of the head/neck complex.

To model the active muscles, the properties of the elements defined were changed in the file generated by the ANSYS program before using the LS‐DYNA solver to obtain the result file. Three functions expressions to define the muscle tension development are:

(i) active‐length function:

(1) FLx=Fmaxexp−llopt−lCsh2,

(ii) active tension‐velocity function:

(2) Fvv=0for vn≤−11+vn1−Cshortfor−10<vn≤1+vnCmvlCleng1+vnClengfor vn>0  ,

and (iii) activation status time function:

(3) Nat=  Ainit  for t≤Trerflex1+Ainit−1Ta−Tne−TneTa−Tneexpt−TrerflexTa+TneTa−Tneexpt−TrerflexTnefor t>Trerflex.

**Figure 2:**
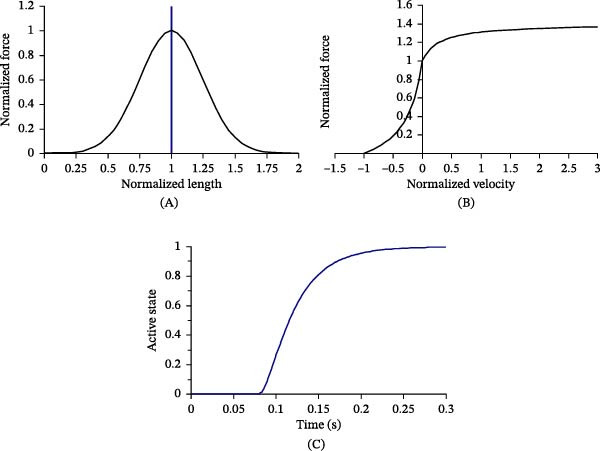
Graphs of functions (A) *F*_*L*_(*L*), (B) *F*_*v*_(*V*), and (C) *N*_*a*_(*t*).

Figure 2 illustrates the characteristic functions used in the muscle model, including the force–length relationship (*F*_*L*_), force–velocity relationship (*F*_*v*_), and the muscle activation function (*N*_*a*_). The activation level (*N*_*a*_) varies between 0 and 1 and evolves with time according to the activation function. The instantaneous muscle force of each muscle is calculated using a Hill‐type muscle formulation, which depends on the maximum muscle force, the activation level, and the force–length and force–velocity relationships. The corresponding parameters used in the LS‐DYNA solver are listed in Table 2.

**Table 2:** Variables to model active muscles.

Spring muscle	*L*_0_ (mm)	*V*_max _ (mm/s)	*F*_max _ (Kg*mm/s^2^)	*L*_max_	*A* (mm^2^)
Sternocleidomastoid	180	1980	44,825	0.8	358.6
Longus colli	60	660	25,000	0.8	200.0
	15	165	25,000	0.8	—
	40	440	25,000	0.8	—
Longus capitis	85	935	50,000	0.8	200.0
	55	605	50,000	0.8	—
Scalenus anterior	110	1210	41,400	0.8	165.6
	80	880	41,400	0.8	—
Scalenus medius	65	715	10,900	0.8	43.6
Scalenus posterior	60	660	34,000	0.8	136.0
Trapezius	160	1760	87,500	0.8	350.0
Semispinalis capitis	120	1320	37,500	0.8	150.0
	90	990	37,500	0.8	—
Semispinalis cervicis	80	880	17,950	0.8	71.8
Longissimus capitis	15	1265	20,000	0.8	80.0
	75	825	20,000	0.8	—
Longissimus cervicis	75	825	20,000	0.8	80.0
Splenius capitis	175	1925	18,700	0.8	224.4
	110	1210	18,700	0.8	—

### 2.3. Ejection Simulation and Boundary Conditions

A dynamic FEA was carried out using LS‐DYNA to compute the cervical spine’s biomechanical response during high‐G pilot ejection. The simulation was designed to replicate the extreme acceleration conditions experienced by pilots during emergency ejection, where the cervical spine undergoes rapid flexion–extension movements due to high vertical forces.

The ejection simulation was conducted by applying a vertical acceleration load to the inferior surface of vertebra T1, mimicking the forces transmitted from the ejection seat to the pilot’s body. The acceleration profile used in the simulation was derived from real‐world ejection scenarios and previous experimental data: (1) Peak acceleration: 10G (98 m/s^2^), (2) maximum acceleration rate: 125 G/s, and (3) total duration: 150 ms. The acceleration curve followed a gradual onset phase, reaching peak acceleration within the first 80 ms, maintaining this level for 70 ms with a total of 150 ms simulation period [30].

To ensure a realistic and controlled simulation environment, boundary conditions were applied to restrict undesired movements while allowing the cervical spine to respond naturally to the applied forces: (1) In order to mimic the upward acceleration of the ejection seat, the T1 vertebra is limited to moving just vertically, avoiding excessive lateral or rotational movement. (2) Head and upper cervical spine: Free to rotate and translate according to the applied forces, allowing for the natural development of flexion–extension motion. (3) Soft tissue interactions: Modeled using nonlinear contact mechanics, ensuring realistic interaction forces between vertebrae, intervertebral discs, and surrounding soft tissues. These boundary conditions reduced computational artifacts and ensured ejection simulation realistically represented the cervical spine’s biomechanical response to high‐G loading [30].

In this study, for each muscle group with tension developed based on a defined Hill‐type muscle, the activation time was varied between 26 and 92 ms to simulate different reflex delays observed in human muscle response to external stimuli during high‐G events. The activation time directly influences the timing of force generation and the magnitude of the force that muscles exert on the cervical spine. Short activation times (26–46 ms) were hypothesized to result in faster muscle response, enabling the muscles to resist excessive motion and stabilize the cervical spine more effectively. Longer activation times (76–92 ms), on the other hand, were expected to cause delayed muscle response, potentially allowing for excessive flexion or extension before the muscles could act to stabilize the cervical spine.

## 3. Results

**Figure 3:**
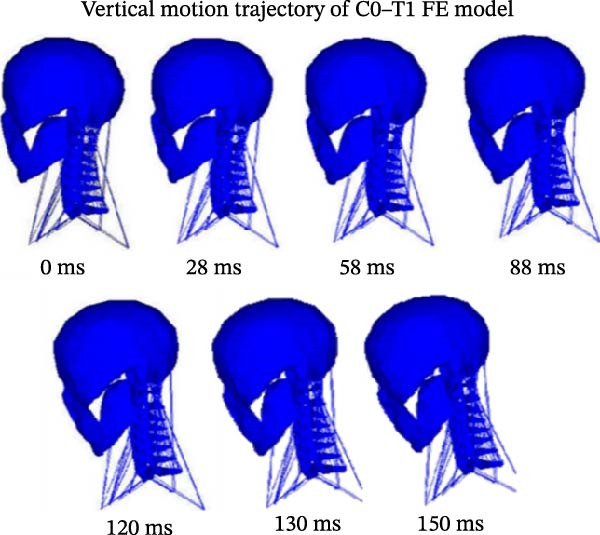
A typical plot of vertical motion trajectories of C0–T1 FE model at different times.

A typical head–neck distorted plots under the influence of active muscles in ejection simulation are displayed in Figure 3. The figure shows different computed deformed plots of the entire C0–T1 complex at different times of the 150 ms simulation ejection solution run process.

### 3.1. Effect of Activation Time on Cervical Rotation Angle

**Figure 4:**
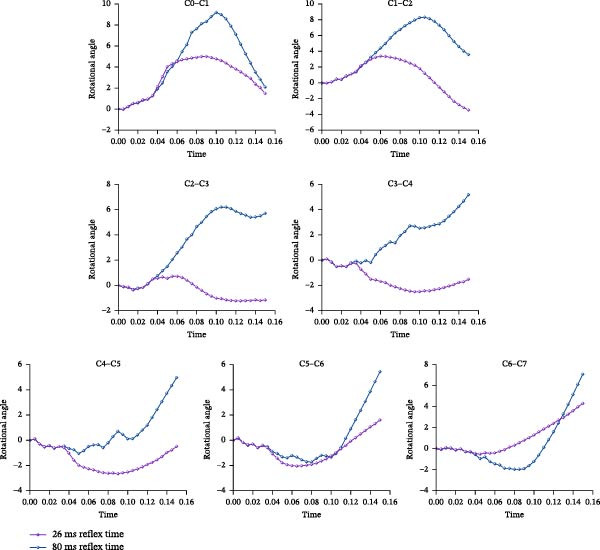
Visualization of rotation angles of each cervical column from C0‒C7 with reflection time of 26 versus 80 ms.

**Figure 5:**
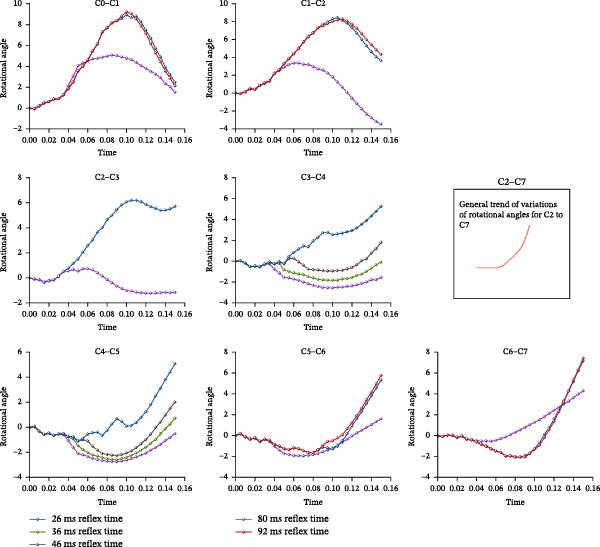
Visualization of cervical sagittal plane rotation angle.

The computed results of the rotational angles at each cervical level of C0–C7 at different activation (reflex) times of (26 ms), and (26, 36, 46, 92 ms), compared to computed results at 80 ms activation time, were shown in Figures 4 and 5, respectively.

From Figure 4, the general trend of the variations of rotational angles of cervical spine columns C0–C1 and C1–C2 were inverted U‐shape, whereas the general trend for the other columns from C2–C7 were U‐shape.

In C0–C1 and C1–C2 segments, there were no intervertebral discs compared to the other segments from C2–C7 levels, the rotational angulation motions depended mainly on the bony geometrical articulation, associated ligaments, and turning moments generated by the attached muscles’ tension developed, resulting in the general inverted U‐shapes. However, the earlier activation of muscle activities had effectively reduced the magnitudes of flexion, with maximum flexion rotation occurred at shorter times. At 150 ms, due to the differences in the rate of decrease in flexion after peak for C0–C1 and C1–C2 levels, the magnitude difference of flexion rotation angulation at C0–C1 was much smaller in comparison to C1–C2, whereby it varied from flexion to extension in the 26 ms activation time.

For levels between C2 and C5, the results showed these levels were mostly in flexion for activation time of 80 ms, while mostly in extension motion for activation time of 26 ms.

For C5–C6 level, under activation time of 26–80 ms, the extension motions were of same magnitude and duration between 0 and 110 ms. However, after 110 ms, the segment went into flexion motion of smaller magnitude for 26 ms activation time. Similar trends of motions were observed for C6–C7 level; duration of about 70–110 ms of extension motion with different maximum values were observed for 26–80 ms activation times, respectively.

Figure 5 shows the motions at different levels under different activation times compared to those at 80 ms activation time. With activation at 90 ms, most levels showed similar trends of flexion/extension of same magnitude and duration compared to those activated at 80 ms, as clearly shown in C0–C2 and C5–C7 levels. For other activation times (26, 36, and 46 ms), drastic changes in motions were observed in C0–C5 levels. C3–C4 and C4–C5 levels underwent extension and flexion motions of different magnitudes and durations, compared to flexion motion throughout for activation at 80 ms. C5–C7 levels showed both flexion and extension motions at all different activation times, with almost same magnitude and duration (about 110 ms) of extension but of different magnitudes of flexion after 110 ms in C5–C6 level. Similar trends were observed in C6–C7 level, but with extension duration of about 70–110 ms of different maximum values for activation times 26–80 ms, respectively.

In general, all levels exhibit either flexion/extension motions or vice versa within the 150 ms simulated time span under different activation times. The rates of change (gradients) of flexion or extension were less steep for earlier activation times, suggesting that faster reflex time could minimize severe neck injury under pilot ejection.

### 3.2. The Overall Effect of Reflex Time on the Cervical Spine

**Figure 6:**
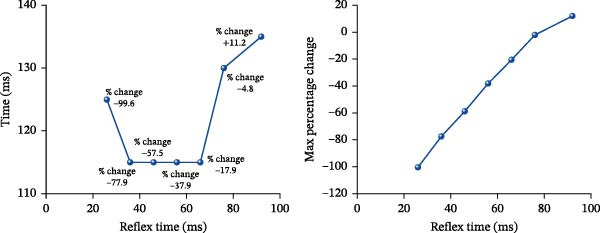
Plots of occurrence time of the maximum percentage change and maximum percentage change of C0–C7 rotation angle against reflex times.

In the previous section, the computed results on the effects of different activation (reflex) times on the motions of individual cervical spine column were discussed. However, to understand the full effect of cervical muscles reflex time on the motion of flex/extend of the whole head/neck complex under simulated ejection, the maximum variations of rotational angles, (in term of percentage (%)) of the head (C0) with respect to C7 was determined with reference to the motion computed at activation time of 80 ms, and plotted as the shown in Figure 6.

From Figure 6, it was observed that the maximum percentage change in rotational angles of C0–C7 for different reflex times were between 115 and 135 ms of the 150 ms simulation process, that is within constant maximum acceleration of 10 G. There was almost a linear reduction in the rotational angles of C0–C7, varied from −4.8% to −99.6%, with increasingly faster reflex time of from 76 to 26 ms; however, for reflex time at 92 ms, there was an increase of 11.2% in the rotational angle of C0–C7.

These findings suggested that the muscle reflex time influenced the increasing or reducing the flexion/extension motions of the head/neck complex under the simulated loading and boundary conditions, offering the potential of minimizing severe neck injury during pilot ejection.

## 4. Discussion

The FEM has a solid reputation as a versatile engineering tool. FE models are frequently used to examine the biomechanical responses of biological structures under various simulated situations, supplementing experimental studies. Prior FE models for dynamic research typically comprised a sequence of vertebrae joined by intervertebral discs and ligaments [51, 52]. These models were unable to offer a thorough interaction between the motion segments and could only forecast the overall response to loads. To mimic the vertebral stress in the cervical spine under ejection, Sadegh and Tchako’s study created a thorough FE model with simplified mechanical properties of different bone components. Nevertheless, the impact of the head on the neck’s reaction was not examined [53, 54]. To depict the entire head–neck complex, an idealized three‐dimensional FE model of the human cervical spine was used in this project. Teo [30] and Zhang previously created this FE model using ANSYS/LS‐DYNA (ANSYS, Inc., Pennsylvania, USA).

The results indicate that activation time significantly affects the rotational motion of the cervical spine during pilot ejection. Shorter activation times (26–46 ms) resulted in a transition from flexion to extension at C3–C4 and C4–C5, with earlier muscle engagement producing stronger stabilizing forces. The duration of this transition was longer for faster reflex times and occurred earlier in the simulation. Conversely, longer activation times (76–92 ms) led to a substantial increase in C5–C7 rotation angles, contributing to excessive hyperflexion, which could increase injury risk. An optimal activation time of ~46 ms helps minimize excessive flexion without inducing hyperextension.

Other studies show that the influence of activation time on intervertebral disc deformation is primarily reflected in how the muscles regulate spinal stability during high‐G conditions [5, 16, 27]. This study shows shorter activation times (26–46 ms) allow early muscle engagement, with smaller range of rotational motions, may aid in maintaining disc alignment and reducing excessive compression forces in the disc of C3–C5 levels. This early stabilization ensures that the mechanical load is evenly distributed across the cervical spine, preventing localized stress accumulation [55]. In contrast, longer activation times (76–92 ms) delay the muscle response, increasing the likelihood of uncontrolled spinal motion. This delay contributes to greater instability in C5–C6 and C6–C7, where the lack of timely muscular resistance may cause excessive disc deformation. The delayed response allows for larger displacement of vertebral segments before stabilization occurs, increasing the risk of disc‐related injuries such as bulging or herniation [56, 57]. The findings suggest that an optimal activation time (~46 ms) enables the muscles to provide sufficient resistance to spinal compression while avoiding excessive stiffening, which could otherwise reduce shock‐absorbing capacity. The relationship between activation timing and intervertebral disc protection underscores the importance of optimizing neuromuscular responses in high‐acceleration scenarios.

To assess the overall impact of reflex time on cervical spine dynamics, rotational angles of C0–C7 were analyzed under different activation delays. The data revealed a uniform reduction in overall flexion–extension movement with shorter activation times, whereas longer reflex times (76–92 ms) resulted in increased overall flexion. Figure 6 illustrates the maximum percentage change in rotational angles of C0–C7 across different activation times. The findings suggest that a reflex time of 26 ms reduces rotational angles by nearly 99.6%, while a reflex time of 92 ms leads to an 11.2% increase in rotation. Although established injury criteria such as ligament strain or spinal cord compression were not explicitly quantified in this study, excessive cervical rotation has been widely used as a surrogate indicator of injury risk in previous biomechanical studies. Therefore, these results highlight the protective role of faster reflex times in minimizing excessive cervical spine motion and potential neck injury during ejection [3, 5, 58]. Understanding the influence of reflex timing on cervical spine motion may help improve protective strategies and injury mitigation approaches in high‐acceleration environments.

It should also be noted that, in the present study, cervical muscle groups were activated simultaneously using a common activation profile while varying only the reflex delay. This modeling strategy does not attempt to reproduce the full complexity of muscle‐specific neuromuscular coordination observed in vivo, where recruitment timing and activation magnitude may differ across muscles and tasks. Instead, the simplified coactivation approach allows the model to represent the overall stabilizing contribution of the cervical musculature during high‐G loading while providing a controlled framework to investigate the influence of neuromuscular response timing on head–neck biomechanics. By reducing variability in muscle recruitment patterns, the model can isolate the effect of reflex delay on cervical spine motion and loading. Similar simplified muscle activation strategies have been adopted in previous finite element studies investigating head–neck biomechanics under dynamic loading conditions [30].

Although this work offers insightful information about the function of activation time in cervical spine biomechanics, it should be noted that it has some limitations. For example, the impact of muscle fatigue from extended high‐G exposure was not considered, which could affect the assessment of long‐term injury risk. In cervical spine biomechanics, more investigation is required to improve the precision and relevance of these results. Important topics for further research include: (1) Refining muscle activation modeling: Incorporating electromyographic (EMG) data to validate and refine muscle activation models, ensuring greater accuracy in simulating real‐time neuromuscular responses [59]. (2) Personalized activation timing analysis: Exploring how individual differences in body composition and muscle strength influence activation time and cervical spine biomechanics, particularly for pilots of varying physiques [60]. (3) Integration with ejection seat cushioning systems: Investigating how activation time interacts with seat impact absorption mechanisms to optimize cervical spine protection during ejection [61, 62]. Addressing these areas will contribute to a more comprehensive understanding of neuromuscular control in high‐G environments, ultimately improving injury prevention strategies and pilot safety measures.

## 5. Conclusions

This study highlights the critical role of activation time in cervical spine biomechanics during pilot ejection. Shorter activation times (26–46 ms) optimize cervical spine kinematics by reducing excessive flexion at C3–C4 and C4–C5, thereby enhancing stability and minimizing injury risk. In contrast, delayed activation times (>76 ms) result in excessive C5–C7 rotation, increasing the likelihood of hyperflexion‐related injuries. To enhance pilot safety, it is recommended that muscle activation strategies be optimized to improve neuromuscular response efficiency under high‐G conditions. The computed articulated segmental motions at different levels provide valuable data for implementing artificial intelligence (AI) assisted training programs and protective devices designed to enhance early synchronization of different major neck muscles engagement to effectively minimize cervical spine injuries in emergency ejection. Future studies should explore real‐time neuromuscular adaptations and develop personalized activation strategies to further improve protection mechanisms in high‐acceleration environments.

## Author Contributions

**Jiongxiang Zhao**: writing – original draft, visualization, software, methodology, formal analysis, investigation, conceptualization. **Zanni Zhang**: writing – original draft, software, methodology, formal analysis, investigation, conceptualization. **Zsolt Radak**: resources, methodology, investigation. **Xuanzhen Cen**: writing – review and editing, methodology, visualization. **Ee-Chon Teo**: resources, methodology, investigation. **Minjun Liang**: writing – review and editing, methodology, investigation. **Yaodong Gu**: writing – review and editing, visualization, supervision, software, methodology, investigation, funding acquisition, conceptualization.

## Funding

This study was funded by Zhejiang Provincial Natural Science Foundation of China for Distinguished Young Scholars (Grant LR22A020002), Zhejiang Provincial Key Research and Development Program of China (Grant 2023C03197), Ningbo key R&D Program (Grant 2022Z196), Research Academy of Medicine Combining Sports, Ningbo (Grant 2023001), National Key Research and Development Program of China (Grant 2024YFC3607305), and Zhejiang Rehabilitation Medical Association Scientific Research Special Fund (Grants ZKKY2023001 and 2023022).

## Conflicts of Interest

The authors declare no conflicts of interest.

## Data Availability

All data relevant to the current study are included in the article, further inquiries can be directed to the corresponding author.
